# Plasma versus serum: which is better for proteomic blood biomarker analysis? Evaluation of the novel NULISA platform

**DOI:** 10.1002/alz70856_106497

**Published:** 2026-01-08

**Authors:** Marissa F Farinas, Yijun Chen, Xuemei Zeng, Michel N Nafash, Alexandra Gogola, Julia K. Kofler, Dana L Tudorascu, C. Elizabeth Shaaban, Jennifer H Lingler, Tharick A Pascoal, William E Klunk, Victor L. Villemagne, Sarah B Berman, Robert Sweet, M. Ilyas Kamboh, Milos D. Ikonomovic, Beth E. Snitz, Ann D Cohen, Oscar L Lopez, Thomas K Karikari

**Affiliations:** ^1^ University of Pittsburgh School of Medicine, Pittsburgh, PA, USA; ^2^ University of Pittsburgh, Pittsburgh, PA, USA; ^3^ University of Pittsburgh, School of Medicine, Pittsburgh, PA, USA; ^4^ UPMC, Pittsburgh, PA, USA; ^5^ University of Pittsburgh Alzheimer's Disease Research Center (ADRC), Pittsburgh, PA, USA; ^6^ Department of Psychiatry and Neurology, University of Pittsburgh, Pittsburgh, PA, USA; ^7^ VA Pittsburgh Healthcare System, Pittsburgh, PA, USA; ^8^ School of Public Health, University of Pittsburgh, Pittsburgh, PA, USA; ^9^ Department of Human Genetics, University of Pittsburgh, Pittsburgh, PA, USA

## Abstract

**Background:**

Blood biomarker studies most often use plasma samples. Suitability of serum as an alternative sample type remains unclear, despite many clinical chemistry laboratories preferring it over plasma. We compared the technical performance of the novel NUcleic acid‐Linked Immuno‐Sandwich Assay (NULISA), a blood‐based targeted proteomic biomarker assay, in plasma and serum samples processed from identical blood draws in a memory clinical cohort.

**Methods:**

Paired plasma and serum samples from 43 donors (75.2±7.8 years, 41.9% female, 32.6% probable AD) from the University of Pittsburgh ADRC were analyzed using the NULISAseq CNS disease panel 120 (v2) on an Alamar ARGO^TM^ system, following manufacturer protocols. Protein levels were quantified by next generation sequencing, normalized, scaled, and log 2‐transformed to NULISA Protein Quantification (NPQ) units. Spearman's rank correlation assessed concordance between plasma and serum NPQs, while the Wilcoxon rank‐sum test evaluated differences in protein levels. Receiver operating characteristic (ROC) curves and the area under the curve (AUC) were used to determine diagnostic accuracy.

**Results:**

The assay achieved high analyte detectability (95.7% ± 14.2%) with low variability (%CV: 4.9%). Strong correlations (Spearman rho: 0.75‐0.96) were observed for traditional AD biomarkers (Aβ42, *p*‐Tau217, *p*‐Tau231, *p*‐Tau181, GFAP, NEFL) across both matrices. ANXA5, NRGN, and Oligo‐SNCA were the top three targets significantly more detectable in plasma (log2 plasma vs. serum fold change: 9.72, 9.48, and 4.99), while TIMP3, S100A12, and BDNF were the top three targets that showed higher levels in serum (log2 plasma vs. serum fold change: ‐2.91, ‐2.66, and ‐2.42). Notably, Aβ42, *p*‐tau181, *p*‐tau217, and *p*‐tau231 levels were significantly higher in plasma than in serum (*p* <0.001). Contrarily, GFAP and NEFL levels were similar in plasma and serum (*p* >0.05). Serum ACHE (AUC: 0.874, 95% CI: 0.768‐0.981), plasma IGF1R (AUC: 0.874, 95% CI: 0.760‐0.989), and plasma ACHE (AUC: 0.842, 95% CI: 0.703‐0.982) were the top three non‐traditional targets that distinguished between AD and control individuals.

**Conclusion:**

NULISAseq‐based AD blood biomarkers in paired plasma and serum are highly correlated; however, the absolute levels significantly vary by matrix type. These findings highlight the importance of considering specimen type in clinical study designs to ensure the reliability and accuracy of AD biomarker diagnostics.

